# Mixed-precision iterative refinement using tensor cores on GPUs to accelerate solution of linear systems

**DOI:** 10.1098/rspa.2020.0110

**Published:** 2020-11-25

**Authors:** Azzam Haidar, Harun Bayraktar, Stanimire Tomov, Jack Dongarra, Nicholas J. Higham

**Affiliations:** 1 NVIDIA, Santa Clara, CA, USA; 2 Department of Electrical Engineering and Computer Science, University of Tennessee, Knoxville, TN, USA; 3 Computer Science and Mathematics Division, Oak Ridge National Laboratory, Oak Ridge, TN, USA; 4 Department of Mathematics, University of Manchester, Manchester M13 9PL, UK

**Keywords:** half precision arithmetic, mixed precision solvers, LU factorization, iterative refinement, GMRES, GPU computing

## Abstract

Double-precision floating-point arithmetic (FP64) has been the de facto standard for engineering and scientific simulations for several decades. Problem complexity and the sheer volume of data coming from various instruments and sensors motivate researchers to mix and match various approaches to optimize compute resources, including different levels of floating-point precision. In recent years, machine learning has motivated hardware support for half-precision floating-point arithmetic. A primary challenge in high-performance computing is to leverage reduced-precision and mixed-precision hardware. We show how the FP16/FP32 Tensor Cores on NVIDIA GPUs can be exploited to accelerate the solution of linear systems of equations *Ax* = *b* without sacrificing numerical stability. The techniques we employ include multiprecision LU factorization, the preconditioned generalized minimal residual algorithm (GMRES), and scaling and auto-adaptive rounding to avoid overflow. We also show how to efficiently handle systems with multiple right-hand sides. On the NVIDIA Quadro GV100 (Volta) GPU, we achieve a 4×−5× performance increase and 5× better energy efficiency versus the standard FP64 implementation while maintaining an FP64 level of numerical stability.

## 1. Introduction

A fundamental requirement in scientific computing is the ability to solve a system of linear equations

1.1 Ax=b,

where *A* is a large, dense *n* × *n* non-singular matrix. This can be done at a speed that is close to the peak performance on current computer architectures, for example, by using libraries such as the NVIDIA cuSolver library [1], MAGMA [2,3] and MKL [4] that redesign and highly optimize the standard LAPACK algorithms [5] for GPU and multi-core architectures. The solvers use direct methods in a fixed/working precision arithmetic, namely, the IEEE standard double-precision 64-bit floating-point arithmetic (FP64), or single precision 32-bit floating-point arithmetic (FP32). Recently, various machine learning and artificial intelligence neural network applications increased the need for half precision arithmetic, and vendors started to accelerate it in hardware, in the form of either the IEEE FP16 format [6] or the bfloat16 format [7] (table 1). Currently, the NVIDIA V100 Tensor Cores (TCs) can execute FP16-TC at up to 120 teraFLOP s^−1^ versus 7.5 teraFLOP s^−1^ for FP64 and 15 teraFLOP s^−1^ for FP32. Thus, developing algorithms to exploit the much higher performance that lower-precision arithmetic offers can have a significant impact in scientific, high-performance computing (HPC).

**Table 1.** Parameters for the IEEE FP16, FP32 and FP64 arithmetic precisions, and their respective peak performances on an NVIDIA V100 GPU. ‘Range’ denotes the order of magnitude of the smallest subnormal (*x*_min,*s*_), and largest and smallest positive normalized floating-point numbers. In comparison, the FP16-TC inputs are FP16, while the outputs and the computations are performed in full (FP32) precision, and the peak performance is 120 Tflop s^−1^.

		range				
arithmetic	size (bits)	*x*_min,*s*_	*x*_min_	*x*_max_	unit roundoff	peak Tflop s^−1^ (V100 GPU)
BFloat16	16	9.2 × 10^−41^	1.2 × 10^−38^	3.4 × 10^38^	3.9 × 10^−3^	n.a.
FP16	16	6.0 × 10^−8^	6.1 × 10^−5^	6.6 × 10^4^	4.9 × 10^−4^	30
FP32	32	1.4 × 10^−45^	1.2 × 10^−38^	3.4 × 10^38^	6.0 × 10^−8^	15
FP64	64	4.9 × 10^−324^	2.2 × 10^−308^	1.8 × 10^308^	1.1 × 10^−16^	7.5

This paper presents a class of mixed-precision algorithms and an accompanying set of computational techniques that we have developed to accelerate (1.1) in FP64, which is the de facto standard for scientific computing. We show that the new mixed-precision techniques can accelerate the solution by a significant factor using the faster lower precisions, while still retaining FP64 quality.

The mixed-precision iterative refinement algorithm computes an LU factorization of *A* in low precision, uses the LU factors to compute a initial approximation *x*_0_ and then carries out an iterative refinement process in FP64 arithmetic. The refinement process repeatedly solves the correction equation *Ac*_*i*_ = *b* − *Ax*_*i*_ for *c*_*i*_ then updates the solution through *x*_*i*+1_ = *x*_*i*_ + *c*_*i*_, continuing until *x*_*i*+1_ has a backward error at the FP64 level, or some other user-specified tolerance. These methods have been studied in the past, as discussed in §2. A persistent challenge has been to redesign the techniques for new architectures and to develop highly tuned implementations that resolve computational issues such as inefficient parallelization, scaling and use of mixed-precision calculations. To address this problem on GPU TCs, we develop a number of innovations for mixed-precision computations (outlined in §3) as well as leverage building blocks from HPC numerical libraries such as cuSolver and MAGMA, which provide state-of-the-art, high-performance algorithms such as LU factorization—including a set of highly tuned mixed-precision iterative refinement algorithms using either the FP32 or the FP16 as lower precision for the LU factorization (e.g. FP32 → FP64 and FP16 → FP64) [8,9].

## 2. Related work

Iterative refinement is a long-standing method that was programmed by Wilkinson in the 1940s for the ACE digital computer. The idea is to improve the computed solution of a linear system by iteratively solving a correction equation and adding the correction to the current solution; see Wilkinson [10], Moler [11], Stewart [12], Demmel [13] and, for a comprehensive treatment, Higham [14, Chap. 12]. The three tasks—original solve/factorization, residual computation and correction equation solve—can be done in the same precision (fixed-precision) or in different precisions (mixed-precision). Fixed-precision iterative refinement was analysed by Skeel [15] for a solver based on LU factorization and by Higham [16,17] for a general solver. In the 2000s, motivated by processors equipped with FP32 that had speed 2× that of FP64, mixed-precision iterative refinement—with the LU factorization done in FP32 and everything else done in FP64—was explored in [18,19].

Replacing the direct triangular solves of the correction equation with an iterative method, as suggested in [20] in a mixed-precision context, leads to ‘nesting’ of two iterative methods, which in general are called ‘inner–outer’ iterations, the latter having been studied both theoretically and computationally [21–23], including in mixed-precision computation scenarios [24]. Recently, Carson & Higham [20,25] analysed a three-precision iterative refinement scheme (factorization precision, working precision, residual precision) and concluded that if the condition number of *A* is not too large, namely $\kappa_{\infty }(A)=||A|{|}_{\infty }||A^{-1}|{|}_{\infty }<10^{4}$, then using FP16 for the *O*(*n*^3^) portion (the LU factorization) and (FP32, FP64) or (FP64, FP128) as the (working, residual) precision for the *O*(*n*^2^) portion (refinement loop), one can expect to achieve forward error and backward error on the order of 10^−8^ and 10^−16^, respectively. We note that, if $\hat{x}$ is an approximate solution of *Ax* = *b* the forward error is defined by $||\hat{x}-x|{|}_{\infty }/||x|{|}_{\infty }$ and the backward error is defined by $\min\{\epsilon :(A+\Delta A)\hat{x}=b,\;||\Delta A|{|}_{2}\leq \epsilon ||A|{|}_{2}\}$ and can be evaluated as $||r|{|}_{2}/(||A|{|}_{2}||\hat{x}|{|}_{2})$, where $r=b-A\hat{x}$. Carson and Higham also proposed the use of the GMRES method preconditioned by the FP16 LU factorization as the refinement procedure and showed that in this case the constraint on the condition number can be relaxed to *κ*_∞_(*A*) < 10^8^ when the (working, residual) precision is (FP32, FP64) and to 10^12^ when the (working, residual) precision is (FP64, FP128). Analysis covering this GMRES-based approach when two precisions are used with the residual precision equal to the working precision is given in [26].

## 3. Contributions

The primary motivation of this paper is to develop an HPC framework for mixed-precision Tensor Core Accelerated Iterative Refinement solvers (tcairs) that use FP16-TC. To this end, we make the following contributions.

— We develop a framework for mixed-precision solvers as well as TCs-enabled dense linear algebra building blocks that can be used to exploit the FP16-TC in HPC applications.
— We provide algorithmic advancement to increase the solvers’ applicability to matrices not representable in FP16’s limited range. In our algorithm, the original data is never represented in precision below FP32. This technique is the first step toward our proposition for a new set of mixed-precision factorization algorithms. In addition, we investigate scaling techniques that can also help fix issues related to the FP16 range.
— We introduce a new class of multiprecision factorization algorithms. Iterative refinement solvers have always used only one precision for the factorization and a higher precision for the refinement loop, e.g. factorization in FP16 and a refinement loop in FP64. In our work, the factorization itself is implemented in multiprecision to allow better accuracy and to be able to solve a wider range of problems than previously possible.
— We develop a performance model to accurately predict performance gains, allowing users to decide in advance if iterative refinement solvers can be beneficial/applicable for their problems.
— We present a range of problems from different application areas, both dense and sparse, that we show to be accelerated up to 5× when using the FP16-TC, or about 2× when using the FP32 arithmetic.
— We show how the performance of the mixed-precision iterative refinement solver is not sensitive to the FP64 compute throughput.
— We show how to adapt the solver to efficiently handle linear systems with multiple right-hand sides.
— We develop highly optimized mixed-precision solvers supporting real as well as complex data.
— We present a study of the energy efficiency of the iterative refinement solver exploiting the Tensor Cores and show that it can reduce energy consumption by up to 5 times.

The developments are released in the Cuda toolkit cuSolver library [1] and Magma [2,8].

## 4. Iterative refinement solver: background

The standard method for solving a linear system *Ax* = *b* with an *n* × *n* matrix *A* is Gaussian elimination with partial pivoting, or equivalently, the LU factorization method with partial pivoting. An LU factorization represents *A* as the product of a lower triangular matrix *L* and an upper triangular matrix *U*, so that *A* = *LU*. Thus, solving *Ax* = *b* reduces to solving two triangular systems:

Ax=b⇒LUx=b:  solve Ly=b then solve Ux=y.

The factorization costs *O*(*n*^3^) operations while the triangular solve costs *O*(*n*^2^) operations, so for large *n* almost all the time is spent in the factorization and the triangular solve time is negligible. In practice, partial pivoting is used to ensure numerical stability, resulting in a factorization *PA* = *LU*, where *P* is a permutation matrix. For simplicity of exposition, we suppress *P* in what follows (thus A←PA). Throughout the paper *A* is assumed to be non-singular.

The basic way to solve such a system is to perform these operations in one precision, typically the precision to which the user wishes to obtain the solution, or the precision in which the input data is given. We call this the working precision and denote it by **u**^**w**^.

### (a) Iterative refinement

Iterative refinement aims to improve the accuracy or backward error of a computed solution $\hat{x}$ to *Ax* = *b*. It consists of a series of iterations (the refinement loop) and is described in algorithm 4.1 in the general three-precision form proposed by Carson & Higham [25]. The three precisions of arithmetic used are defined by their respective unit roundoffs:

— **u**^**w**^: the precision at which the data *A*, *b* and the solution *x* are stored.
— **u**^**f**^: the precision at which the factorization of *A* and the correction on step 2 are computed.
— **u**^**r**^: the precision at which residuals are computed on step 1.

The precisions satisfy **u**^**r**^ ≤ **u**^**w**^ ≤ **u**^**f**^. We write ‘in precision u*’ to mean ‘in floating-point arithmetic of precision u*’.


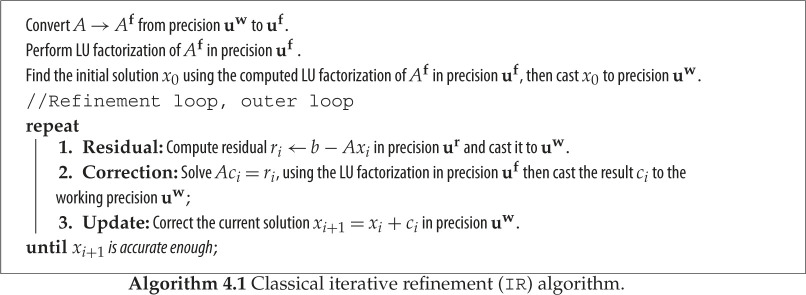


As seen in algorithm 4.1, step 1 is carried out in precision **ur**. Step 2 is performed in the precision **u**^**f**^, which is the precision of the LU factorization: it uses the LU factors to solve the correction equation *Ac* = *r* and then it casts the solution *c*_*i*_ to the working precision if **u**^**w**^. Step 3 is carried out with precision **u**^**w**^. If all three steps can be computed exactly then the iterative refinement algorithm completes in one iteration. However, in floating-point arithmetic the above steps usually need to be repeated.

If **u**^**f**^ = **u**^**w**^ = **u**^**r**^ the method is called fixed-precision iterative refinement; otherwise, it is mixed-precision iterative refinement. Fixed-precision iterative refinement can be used to improve the backward error of an LU factorization without a strong stabilizing pivoting strategy [15,27,28]. On the other hand, mixed-precision iterative refinement also improves the forward error to the working precision if the condition number of *A* is not too large: **u**^**f**^*κ*_∞_(*A*) ≤ 1. We denote this method by IR.

In our analysis and experiments we take **u**^**r**^ = **u**^**w**^, so we do not use extra precision in forming the residuals.

### (b) The LU factorization

**Figure 1.**
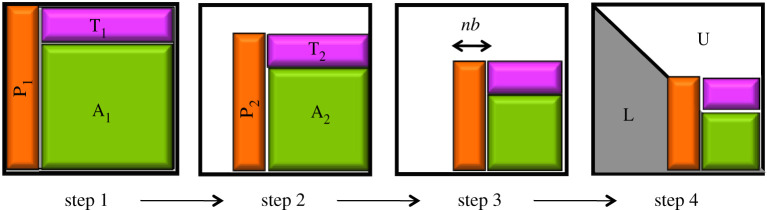
Schematic representation of the LU factorization process.(Online version in colour.)


**Figure 2.**
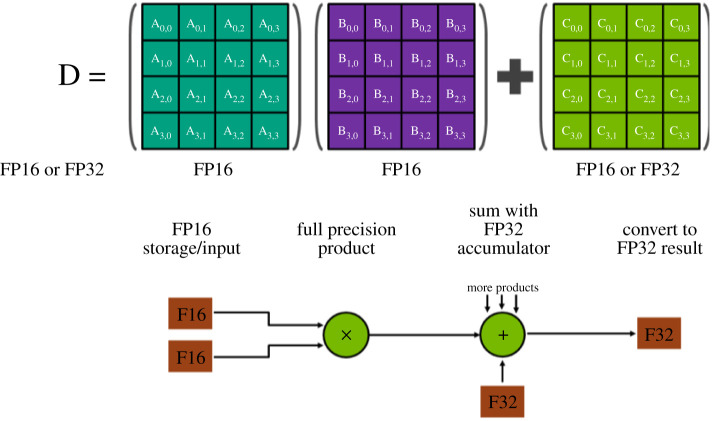
Tensor Core matrix multiply and accumulate, showing how products of scalars within a 4 × 4 matrix multiplication are formed and accumulated. (Online version in colour.)

Algorithmically, as presented in algorithm 4.2 and illustrated in figure 1, LU factorization can be viewed as a sequence of steps with two distinct phases per step: (1) a panel factorization that affects the data depicted by the orange portion of figure 1, and (2) a triangular solve that updates data represented by the magenta portion (denoted by *T*_*i*_) and (3) a trailing matrix update (Schur update) denoted by *A*_*i*_ and represented in green in figure 1. From a software point of view, we know that PanelFactorize is a memory-bound step performed through the Xgetf2 routine and occupies a small portion of the total time, while TrailingMatrixUpdate is compute-bound and is performed using the Level-3 Basic Linear Algebra Subprograms (BLAS) routines Xgemm. The *A*_*i*_ updates occupy the greatest portion of the time spent in the factorization. Thus one might expect the performance of the LU factorization to be asymptotically similar to the Level-3 BLAS Xgemm routine.

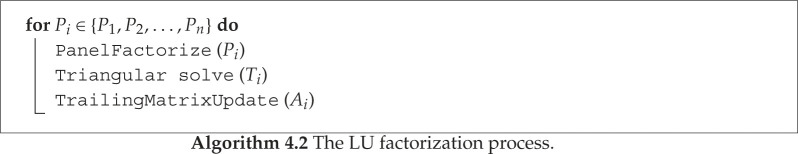


## 5. Multiprecision factorizations

In contrast to the standard factorization that works in one precision (algorithm 4.2), we have developed a new class of multiprecision factorizations that use a higher precision $u^{f_{h}}$ for numerically critical parts of the algorithm, and a lower precision $u^{{\text{f}}_{\text{l}}}$ for the other parts, the parts that are numerically less sensitive and also the most time consuming.

In our previous work [29], we found that performing the factorization in fixed FP16 precision (**u**^**f**^ of algorithm 4.1 equal to FP16) suffers from some issues, notably that the entries of the matrix *A* can be outside the representable range of FP16, potentially making the rounded matrix singular in **u**^**f**^, as well as causing numerical troubles due to a relatively inaccurate panel computation. We then proposed the multiprecision factorization method where we suggested to keep the fast low precision (FP16) for only the ‘most time consuming’ and ‘numerically less sensitive’ portion of the factorization process (e.g. the Schur complement update) and to use a higher precision (FP32) for the critical portion (e.g. the panel portion). In our study, $u^{f_{l}}$ corresponds to FP16 and $u^{f_{h}}$ corresponds to FP32. This is shown in algorithm 5.1.

In algorithm 5.1, the panel factorizations, which cost a total of *O*(*n*^2^) flops, are done in FP32. The Schur complement updates, which cost a total of *O*(*n*^3^) flops, are done as mixed-precision Xgemms, where *P*_*i*_**f**_**l**_ and *T*_*i*_**f**_**l**_ are of lower precision $u^{f_{l}}=FP16$, while the inout *A*_*i*_**f_h_** is of higher precision $u^{\text{f\_h}}=FP32$. This type of operation is a feature of the NVIDIA Tensor Cores hardware, and we are taking advantage of it to provide a fast factorization while maintaining the sensitive portion of the computation in higher precision. For the sake of completeness, we briefly describe how the NVIDIA Tensor Cores operate and allow us to keep *A*_*i*_ in higher precision $u^{\text{f\_h}}=FP32$. The Tensor Cores are programmable matrix-multiply-and-accumulate units that can deliver up to 120 teraFLOP s^−1^ on NVIDIA Volta GPU hardware. This number is higher on newer NVIDIA hardware. On an NVIDIA Volta GPU, each Tensor Core performs the operation D=A×B+C, which is an ‘FP16 input using a full-precision product and FP32 accumulate’ as shown in figure 2. The matrix inputs A and B are in FP16, while the matrices C and D could be in FP16 or FP32. In our case the matrix D is the same matrix as C and it is in FP32.

By using the multiprecision factorization, we can maintain the benefit of the high speed of the FP16 precision while producing a better quality factors *L* and *U* than if we perform the whole factorization in fixed FP16.

We emphasize that the two precisions used here in the multiprecision factorization are not to be confused with the different precisions (**u**^**w**^, **u**^**r**^, **u**^**f**^) used in the refinement process. From the refinement point of view, the factorization is associated with one precision **u**^**f**^ in algorithm 4.1 and how it is implemented (fixed or multiprecision) is considered a black box. This type of multiprecision factorization was first developed and released within the Magma library [2,8] and the Cuda toolkit cuSolver library [1].


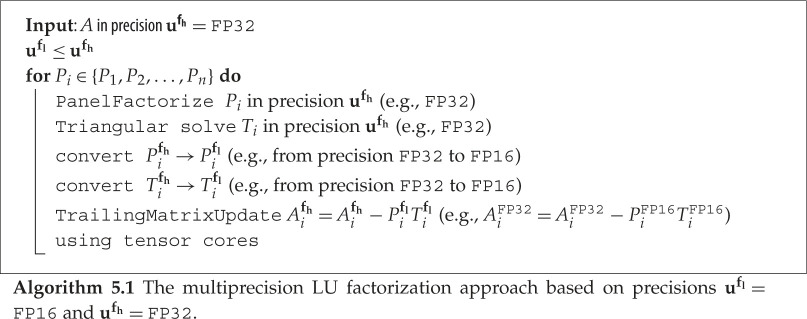


The benefit of the multiprecision factorization is twofold. First, it helps solve many of the FP16 numerical issues, as the input matrix *A* is never converted to FP16, thus there are no issues related to the conversion of *A*. Second, all the sensitive portions of the computation (panel factorization and triangular solve) happen in FP32, thus there are no issues related to FP16 underflow or overflow in these portions. It is only during the trailing matrix update of *A*_*i*_ where FP16 is involved,

5.1 $$C_{FP32}=C_{FP32}-A_{FP16}B_{FP16}.$$

This means that issues related to the FP16 could only appear here if entries of $A_{FP16}$ and $B_{FP16}$ exceed the FP16 range. Thus, we propose an auto-adaptive rounding mode to overcome this issue.

### (a) Auto-adaptive rounding

We propose an adaptive rounding technique that rounds *A* and *B* to nearest FP16 values, except for values larger than the FP16 range which are rounded to the nearer of the maximal or minimal normalized FP16 values (namely ±65 504). Thus, our proposed adaptive rounding method avoids breakdown in the algorithm caused by overflows creating infinities.

As a result, our proposed multiprecision factorization ensures that during the factorization process as soon as the values are within the FP32 range, there will be no overflow related to the FP16. In practice, mixed-precision factorization gives accuracy between that of a fixed FP32 precision factorization and a fixed FP16 factorization. When the fixed FP16 precision factorization works, mixed precision can still produce a backward error between one and two orders of magnitude smaller than the pure FP16 factorization [30], while retaining the high performance of the FP16 variant.

## 6. Iterative refinement with preconditioned GMRES (IRGM)

GMRES [31] is a popular Krylov subspace iteration for solving a general linear system of equations. Following Carson & Higham [20], we consider another variant of iterative refinement by using preconditioned GMRES to approximately solve the correction equation *Ac*_*i*_ = *r*_*i*_ in step 2 of the classical IR in algorithm 4.1. GMRES will be preconditioned by the low-precision LU factors. The idea is that the GMRES solver will provide a better and more stable approximate solution to *Ac*_*i*_ = *r*_*i*_ than the basic triangular solve, which is directly affected by the quality of the low-precision LU factors. Using GMRES, we can still guarantee that the solution of the correction equation *Ac*_*i*_ = *r*_*i*_ has some correct digits and a residual at the level of the convergence tolerance requested by the algorithm. The convergence tolerance is chosen to be of the order of the unit roundoff of the low-precision arithmetic used during the factorization (e.g. we use 10^−4^ or 10^−8^ when the LU factorization is in FP16 or FP32, respectively). We denote this variant by IRGM, and it is described in algorithm 6.1. Note that *U*^−1^ and *L*^−1^ are never explicitly formed; instead matrix–vector products *U*^−1^*L*^−1^*Ay* needed by GMRES are computed by multiplication by *A* followed by two triangular solves. Since this paper focuses on the practical usage and possible performance gains rather than error analysis, we point the reader to [20,25,26] for detailed error analysis of the IR and IRGM techniques.


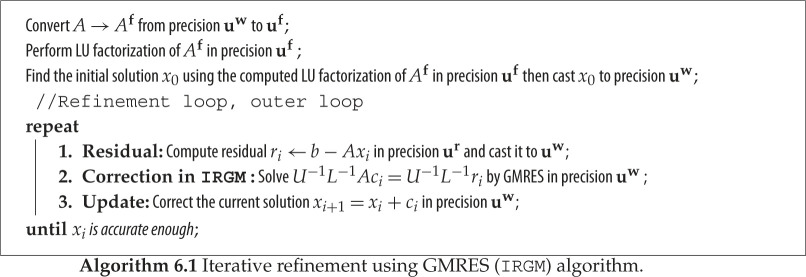


## 7. Preconditioned GMRES

The idea here is to use a preconditioned, full GMRES to solve the original linear system *Ax* = *b*, instead of using preconditioned GMRES to solve the correction equation as in §6. This idea can be viewed as setting the tolerance of the GMRES in §6 to the unit roundoff of FP64, thus the correction will be solved down to FP64 accuracy, meaning the refinement loop of algorithm 6.1 will finish in one outer iteration. This proposition comes from the analysis of the Krylov subspace on which the GMRES method is based. In §6, the refinement loop can be viewed as a restarted GMRES, but instead of performing *m* iterations before each restart, it performs any number of iterations until it reaches the tolerance set for the GMRES (e.g. 10^−4^ in this study), while the method proposed here uses a full GMRES until convergence. It is well known that a full GMRES most likely converges faster than the restarted one because it uses all the previously constructed Krylov subspace to find the new direction to minimize the residual, while the restarted one starts constructing a new Krylov subspace after each restart. Our experiments show that for hard cases, where the convergence requires more than 30 iterations, the proposed preconditioned full GMRES has an advantage over the method proposed in §6, while for problems where the number of iterations is small, both methods behave the same within one to two iterations difference. This is numerically illustrated in §14. On hard cases the classical IR cannot converge, while the IRGM and GM refinement methods initially converge similarly (e.g. cf. figure 7). After that the GM refinement method continues to decrease the residual using the entire previously generated Krylov subspace to find the new direction, while the IRGM method starts the construction of a new set of Krylov subspace vectors, resulting in slower convergence. We mention that for all other cases depicted in §14 both the GM and the IRGM refinement methods behave similarly, while the IR method can fail sometimes. However, we caution that one does not normally run Krylov methods to convergence to the working precision and the convergence rate of GMRES is not well understood for general matrices *A*, so the behaviour we have observed may not always hold.

## 8. Scaling techniques

Numerical algorithms that deal with FP16 computations must deal with the limited range of representable elements in FP16 (see table 1 for more details). For example, when converting a matrix *A* to $A_{FP16}$, the matrix entries may overflow or underflow. Our approach avoids overflow (as long as the FP32 range is not exceeded) and reduces the chance of underflow. Thanks to the proposed innovative multiprecision factorization *A* is never converted to FP16. Instead, it is converted to FP32 which, together with the use of auto-adaptive rounding method (see §5a) in the factorization, excludes such problems. Even though the proposed multiprecision factorization alleviates many of the FP16 numerical issues (e.g. related to conversion or to computation), some other issues still need attention. In particular, if the computation involves values close to or above the range limit then the quality of the LU factors might be compromised and might require other techniques to cope with the low bits that FP16 explores. One such technique is scaling. We note that bfloat16 arithmetic provide a range similar to the FP32 but with short mantissa and thus it could also be used for the factorization and avoids (as long as the FP32 range is not exceeded) overflow related to the FP16 range. However, we expect the short mantissa to be a limitation.

Originally, scaling was introduced in the context of iterative methods to speed up their convergence. Later, scaling was also used in direct methods (both dense and sparse) as a way to reduce the condition number of a matrix. In our work, we introduce scaling in the context of the iterative refinement solver. The goal of scaling is to adjust the range of the data so that the risk of underflow and overflow in computations in lower precision is minimized, and it can also reduce the condition number of the preconditioned matrix in GM and IRGM. Different approaches for scaling could be used. Research about scaling techniques for dense and sparse system can be found in [32,33]. More recently, [34] studied different scaling techniques for the iterative refinement solver using FP16 precision with tests on relatively small matrices. In this paper we study three approaches for scaling proposed in [34] and we show the effect of each method on the convergence of the iterative refinement solver. We first discuss a scalar scaling technique and then we discuss a two-sided diagonal scaling method. We also study a technique that uses a combination of the two scaling approaches.

### (a) Scalar scaling

The idea here is to scale the matrix in such a way that its elements are mapped to be within a constant of the FP16 interval range, i.e. $a_{ij}^{\left(scaled\right)}\in [-\theta x_{\max},\theta x_{\max}]$, where *θ* ∈ (0, 1] is a parameter. (Recall that *x*_max_ is given in table 1.) This technique is described in algorithm 8.1. The strategy does two things: it ensures that the matrix fits into the FP16 range and it stretches the elements to cover a fraction *θ* of the range, so as to make the best possible use of the limited range. Since the matrix is subsequently LU factorized, it is important that some headroom is allowed for growth during the factorization: if the elements grow by a factor *ρ*_*n*_ then *θ* should be less than 1/*ρ*_*n*_. For many problems, *ρ*_*n*_ < 100 with partial pivoting, so *θ* = 0.01 will be sufficient.

One of the effects of this algorithm is that it can result in many elements underflowing, which is possible if many of the elements of *A* are sufficiently smaller in magnitude than *a*_max_.

The effect of this scaling on the quality and the convergence of the iterative refinement solver is analysed in §14.


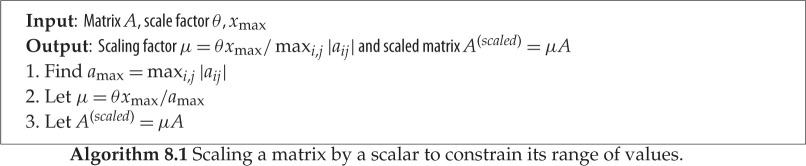


### (b) Two-sided diagonal scaling

To address the issues of the scalar scaling, let us study another scaling strategy that applies a two-sided diagonal scaling, giving *A*^(*scaled*)^ = *R A C*, where $R=\mathrm{diag}(r_{i})$ and $C=\mathrm{diag}(c_{i})$ are diagonal matrices. Note that *r*_*i*_ and *c*_*i*_, *i* = 1:*n* are the scaling factors for each row and column of *A*, respectively. Such scaling algorithms have been developed in the context of linear systems, and in particular we focus on the scaling algorithm provided by LAPACK through the routine Xgeequ. We describe the two-sided diagonal scaling algorithm of Xgeequ in algorithm 8.2. This technique computes row and column scaling intended to equilibrate a matrix *A* and reduce its condition number. *R* holds the row scale factors and *C* the column scale factors, chosen to make the largest element in each row and column of the matrix *A*^(*scaled*)^ (with elements *A*^(scaled)^(*i*, *j*) = *R*(*i*)*A(*i*, *j*)*C(*j*)) have an absolute value of 1 (note that every row and column must be non-zero since *A* is non-singular).


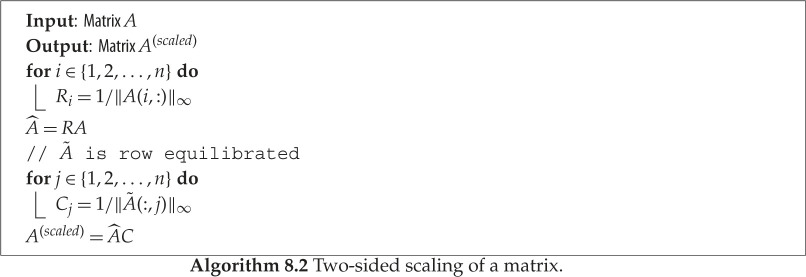


### (c) Two-sided diagonal scaling followed by scalar scaling

The idea here is to use a combination of the scaling techniques described above. We represent the implementation in algorithm 8.3. First, a two-sided scaling will be performed on *A* to smooth its values and reduce its condition number by making all the values within the FP16 range. Then, a scalar scaling will be applied (see lines 2–4 from algorithm 8.3) in order to move the elements towards the largest representable number so as to make the best possible use of the limited FP16 range. More details about this technique can be found in [34]. We note that since the intent of the two-sided diagonal scaling is to make the maximal absolute value of $\tilde{A}$ less than or equal to 1, we can say that *β* in line 2 of algorithm 8.3 will be equal to 1. Regarding the choice of *θ*, if *θ* is close to 1 then we maximize the use of the FP16 range and thereby reduce the chance of underflow (which in the worst case could make the matrix singular). On the other hand, *θ* needs to be sufficiently less than 1 to allow headroom so subsequent computations do not overflow. We take *θ* = 0.1, as in [34].


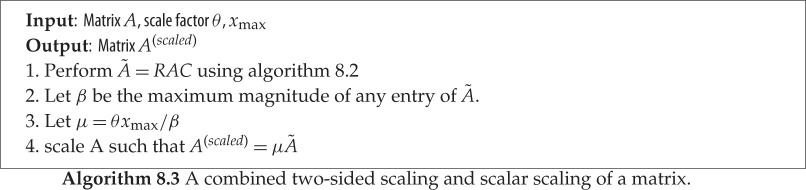


We discuss the three scaling techniques described in §14 and show the benefits of each.

## 9. Multiple right-hand side optimizations

Many engineering applications require *Ax* = *b* to be solved for multiple right-hand sides (RHSs). For that, and in order to accommodate such applications, we designed and developed our tcairs in such a way that it allows for the efficient solution of multiple RHSs. The tcairs consists of two main phases, the factorization phase and the refinement phase. The factorization phase performs an LU decomposition on the matrix *A* and is thus independent of the number of RHSs for which the linear system needs to be solved. As a result, this phase remains the same. The refinement phase is where the solution is refined to achieve the precision of the input data. In this work, we optimized and used the IR method described in algorithm 4.1 for the solution of multiple RHSs. When multiple RHSs have to be solved, we need to adapt the computation of the residual to guarantee that all the solution vectors are accurate. This can be achieved if the error norm used for convergence checking is taken to be the maximal norm of all residuals (e.g. the maximal norm of each residual corresponding to each vector). This way, once the convergence is achieved, we guarantee that all solution vectors are accurate to the tolerance requested. However, since the refinement phase is memory bound, performing the refinement vector-by-vector is too expensive and will lead to a quick drop in the performance. Instead, we replace the matrix-vector operation Xgemv when computing the residual of *b* − *Ax*_*i*_ at iteration *i* with a matrix-matrix product Xgemm to compute all the residuals at once. We also replace the computation of an approximate solution using the LU factorization with the computation of all approximate solutions at once (e.g. replacing the two triangular solves over one vector using Xtrsv routines with two triangular solves for many vectors at once using the trsm routine). Moreover, to avoid computing the maximal norm of each residual, and then take the maximum of all of them, we only compute the norm of the first vector, and check if it satisfies the convergence error tolerance; if not, then we do not compute the remaining residual norm, but rather we step to the next iteration of the refinement loop, and keep doing the same process until that norm (norm of the first residual vector) satisfies the convergence error tolerance. It is at this point that we move to computing the next residual norm and follow the same process until all norms satisfy the convergence tolerance. To summarize, as soon as a residual norm does not satisfy the tolerance, we skip the norm computation of the remaining residuals and perform a new refinement iteration. This technique reduces the amount of memory-bound operations to be performed when multiple RHSs are requested.

## 10. Sensitivity of performance to FP64 compute throughput

Because of the way the mixed-precision IR solver solves a problem—first using reduced and mixed-precision, and then by refining the solution to attain the FP64 accuracy with a memory bandwidth bound algorithm—it highlights the fact that it could be a good candidate to solve an FP64 problem with good performance on a GPU with Tensor Core capabilities, high memory bandwidth and not necessarily high FP64 throughput. This is theoretically demonstrated in detail in §12 through the development of a model of the solver. We also then conducted experiments using the NVIDIA Turing GPU, which is almost identical to Volta GPU except for FP64 compute throughput. This was chosen as a surrogate method to throttling only the FP64 compute throughput on Volta GPUs. We will show in §15 (figure 13) that our tcairs exhibits on a Quadro RTX8000 (Turing TU102 GPU) the same performance as on a high FP64 compute throughput card such as the Quadro GV100 (Volta GV100 GPU).

However, we should mention that applications in scientific computing typically do not only rely on solving a dense linear system *Ax* = *b*, and even when they need it this is typically one component of the whole application. Generally, domain-specific algorithms, and other linear algebra functionality such as fast Fourier transforms (FFTs), eigenvalues, singular values, least squares, symmetric *LDL*^*T*^ decomposition and matrix-matrix products are commonly used in applications. Thus to use GPU cards with low FP64 compute throughput for HPC scientific applications we will need many other mixed-precision numerical algorithm improvements.

## 11. Energy efficient implementation

Power efficiency in HPC is increasingly becoming a concern. Over the last few decades, the improving performance of HPC systems has come at the cost of increased electrical power consumption. The main concerns are increased power bills, e.g. going beyond affordable budgets, and increased impact on the environment. To help mitigate the power constraints in modern and future HPC systems, different approaches have been investigated. Exploiting both the hardware features and algorithms is an effective solution to achieve power efficiency and to address those energy constraints. In this work we redesigned the solvers, which are typically the most time-consuming kernels in HPC applications, to provide energy-efficient alternatives. While most of the energy efficiency approaches aim to reduce the consumption with a minimal performance penalty, our tcairs improves both the performance and the energy efficiency. Indeed, we show below that by efficiently using the Tensor Core hardware and mixed precision, compared with highly optimized linear system solvers, our solver delivers the same accuracy solution and with more than an 80% reduction in the energy consumption.

Also, our tcairs is a GPU-only implementation, which means it does not use the CPU for any computation. Thus, the CPU is idle, which also adds an advantage to the efficiency that such a solver can provide, in particular because CPUs require significant power consumption while providing significantly less performance. In other words, if CPUs contribute to the computation, their rate of FLOPs/Watt is very low, and thus we are going to observe a decrease in the energy efficiency of the solver. We will show in §16 that our solver can effectively reach 94 gigaFLOP s^−1^ per Watt for FP64 real data and 126 gigaFLOP s^−1^ per Watt for FP64 complex data problems. These results significantly improve and extend previous work based on hybrid algorithms that use both CPUs and GPUs [35].

## 12. Performance analysis

**Figure 3.**
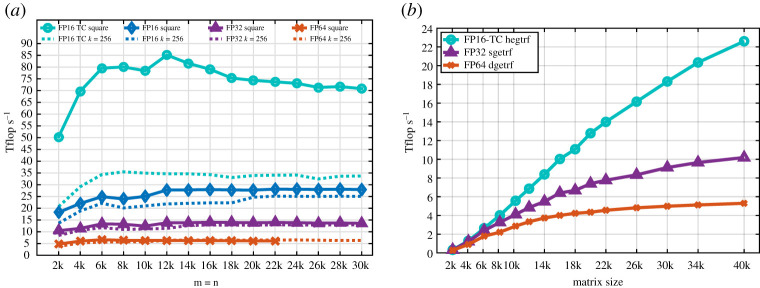
Performance of the LU factorization (the Xgetrf routine) and its main building block (the Xgemm routine) in FP64, FP32 and FP16-TC precisions on an NVIDIA V100 GPU. (*a*) Performance of Xgemm for square matrices and for the rank-k update used in the LU factorization (Xgetrf). (*b*) Performance of Xgetrf for different precisions. (Online version in colour.)

Mixed-precision methods derive their performance from the higher performance of lower-precision arithmetic. The theoretical peaks for the main precisions are shown in the last column of table 1. In practice, the achievable Xgemm performance is less, but maintains a similar trend. Figure 3 quantifies the currently achievable performance for the NVIDIA GV100 GPU. In particular, we see that the PCIe GV100 has a practical peak of 6.8 teraFLOP s^−1^ in FP64, 14 teraFLOP s^−1^ in FP32, 28 teraFLOP s^−1^ in FP16, and a remarkable 85 teraFLOP s^−1^ in FP16-TC (Tensor Cores). However, the performance of the LU factorization relies mostly on the performance of the rank-k Xgemm updates, where the blocking size *k* is typically 256. These updates occur during each step of the LU algorithm, as given in algorithm 4.2. Their performances are shown in figure 3*a* (the dashed lines) for the four available precisions (FP64, FP32, FP16 and FP16-TC). Note that the rank-k hgemm-TC achieves about 35 teraFLOP s^−1^, compared to about 25 teraFLOP s^−1^ for the rank-k hgemm, 13 teraFLOP s^−1^ for the rank-k sgemm, and around 6 teraFLOP s^−1^ for the rank-k dgemm. We note that in our work we always use the FP16-TC (hgemm-TC) and not the standard fused multiply–add (FMA) FP16 hgemm.

The multiprecision LU factorization from algorithm 5.1, with $u^{{\text{f}}_{\text{h}}}$ equal to FP32 and $u^{{\text{f}}_{\text{l}}}$ equal to FP16-TC, was implemented and tuned for current hardware. Figure 3*b* shows the performance for the FP64, FP32 and FP16-TC precisions on GV100 GPUs using the cuSolver library [1]. As expected, the LU implementation follows roughly the same trend as the hgemm-TC kernel for large *n*, showing that the implementation is well optimized and able to attain its theoretical upper bound. The hgetrf-TC solver achieves a speedup of up to 4.5 × over dgetrf and a 2.4× speedup over sgetrf. The speedups further increase for matrix orders larger than 40 000.

Having the achievable performance *P* of the LU factorization, we can derive a theoretical performance analysis of the mixed-precision (MP) algorithms. This analysis is needed in order to understand and predict the cases where iterative refinement can be used. In particular, the interest is in cases where the solution can be reached faster than the reference (e.g. the FP64 dgesv routine). We recall that the iterative refinement solver performs an LU factorization in low precision, followed by a refinement loop based on either the classical IR, the IRGM or the GM refinement method to improve the solution to $\epsilon_{FP64}$. Thus we can model performance for a system of order *n* in the real case by

12.1 $$\text{time for}\;FP64=\frac{2n^{3}}{3P_{dgetrf}}+\frac{2n^{2}}{P_{dtrsv}}$$

and

12.2 $$\text{time for MP }=\frac{2n^{3}}{3P_{hgetrf-TC}}+k\left(\frac{2n^{2}}{P_{dgemv}}+\frac{2n^{2}}{P_{strsv}}+\xi \right),$$

where *P* is the performance of the corresponding routine, *k* is the number of iterations to achieve the FP64 accuracy (including the inner GMRES iterations in the case of the IRGM solver) and *ξ* is other work required by the iterative refinement such as norm computation, residual calculation, pivoting and synchronizations. Experiments show that *ξ* is negligible compared to the dgemv and strsv cost.

**Figure 4.**
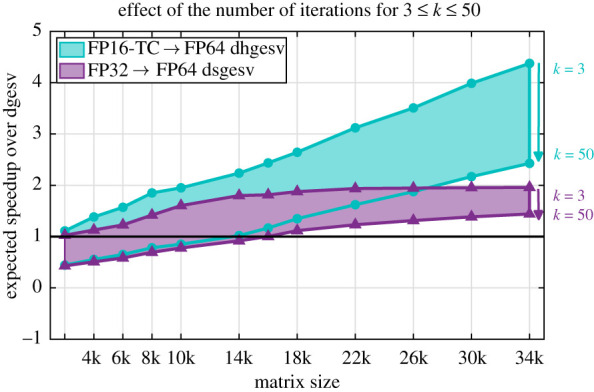
Expected speedup of the iterative refinement solver over dgesv as function of the number of iterations and the matrix size. (Online version in colour.)

Based on the LU performance results provided in figure 3*b* and on the benchmark of the dgemv and strsv routine, we illustrate in figure 4 the expected speedup of the iterative refinement solver using either the FP32 or the FP16-TC precision (dsgesv and dhgesv-TC respectively) as a function of the number of iterations (see also [8] for more details). In the latter case the expected speedup is up to a factor 4. We also note that the study for complex arithmetic is similar and the expected performance follows the same ratio, and for that we omit to redo the same model.

## 13. Experimental set-up

Our experiments were performed on a system with one 10-core Intel(R) Xeon(R) E5-2650 v3 CPU running at 2.30 GHz equipped with an NVIDIA Quadro GV100 PCIe GPU and an NVIDIA Quadro RTX 8000 PCIe GPU.

To study the proposed methods and to highlight their practical use, we performed a large set of experiments on 21 types of matrices, with each type featuring different properties that represent a wide range of real life problems. We found that we could classify the 21 types of matrices using 6 representative cases that we present in table 2. The matrices of singular vectors are random orthogonal matrices from the Haar distribution [36]. In the table, *λ* > 0 denotes that the matrices are symmetric positive definite, that is, they have real, positive eigenvalues.

**Table 2.** Description of the test matrices, where cond is *κ*_2_(*A*).

type	description		
0	—	random numbers with diagonal modified to be dominant	
1	*λ* > 0	random *σ* in $\left[\frac{1}{\text{cond}},1\right]$ such that their logarithms are uniformly distributed	
2	—	random *σ* in $\left[\frac{1}{\text{cond}},1\right]$ such that their logarithms are uniformly distributed	
3	*λ* > 0	clustered *σ*:	$\sigma =[1,\ldots ,1,\frac{1}{\text{cond}}]$
4	—	clustered *σ*:	$\sigma =[1,\ldots ,1,\frac{1}{\text{cond}}]$
5	*λ* > 0	arithmetically distributed *σ*:	$\sigma_{i}=1-(\frac{i-1}{n-1})(1-\frac{1}{\text{cond}}),\;i=1,\ldots ,n$
6	—	arithmetically distributed *σ*:	$\sigma_{i}=1-(\frac{i-1}{n-1})(1-\frac{1}{\text{cond}}),\;i=1,\ldots ,n$
7	*λ* > 0	geometrically distributed *σ*:	$\sigma_{i}={\text{cond}}^{-\frac{i-1}{n-1}},i=1,\ldots n$,
8	—	geometrically distributed *σ*:	$\sigma_{i}={\text{cond}}^{-\frac{n-i}{n-1}},i=1,\ldots ,n$,

We also performed an extensive study on sparse matrices from the SuiteSparse Matrix Collection^1^ [37].

## 14. Numerical behaviour

We first study the numerical behaviour of our tcairs using the three proposed refinement techniques, and show the convergence history of each technique for the different types of matrices. Then we discuss the benefit of using the Krylov GMRES solver. We also show the difference between using the IRGM and the GM technique. We then study the effect of scaling on the convergence of the solver for matrices from table 2 as well as for the matrices from SuiteSparse.

### (a) Convergence behaviour

This study aims to provide an analysis of each method’s sensitivity relative to a wide range of matrices representable for different scientific applications. In addition, our goal is to provide insight into the performance expected from the iterative refinement methods. For example, if an iterative refinement method requires a large number of iterations to achieve FP64 solution accuracy for a certain matrix type, then we can expect that its performance will degrade relative to the standard dgesv, and it may be even slower (see §12 for the expected performance as a function of the number of iterations). We note that the number of iterations that we report is the number of GMRES iterations, which is totalled across all GMRES calls in the case of the IRGM solver. This means that the number of iterations reported is a precise indicator of the time spent in the refinement loop. The convergence criterion used in our experiments is the same as the one used in the state-of-the-art LAPACK iterative refinement solver dsgesv, namely $||Ax-b||/(||A||||x||)<\epsilon^{w}\sqrt{n}$, where *ϵ*^*w*^ is the working precision (e.g. *ϵ*^*FP*64^ in our experiments) and *n* is the size of the matrix.

In the figures below, we plot the relative residual ||Ax−b||/(||A||||x||)

— at each iteration for the FP16-TC variant of the iterative refinement solver using the three proposed iterative refinement algorithms GM, (green) IR (yellow) and IRGM (blue),
— at each iteration for the FP32 variant using the GM algorithm (purple),
— of the final solution at the user level for the standard FP64 solver (horizontal orange line),
— of the final solution at the user level for the iterative refinement solver with the FP16-TC as lower precision (horizontal green, yellow and blues lines for the GM, IR and IRGM refinement methods, respectively).
— of the final solution at the user level for the iterative refinement solver with the FP32 as lower precision (horizontal purple line),

The *no_cvg* text in the figures mean that the corresponding refinement solver did not converge after 200 iterations.

**Figure 5.**
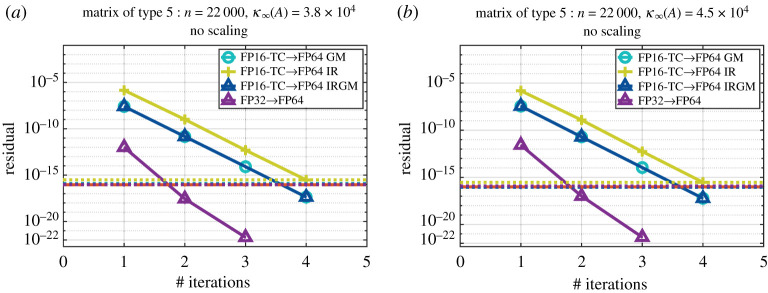
Convergence history of the proposed iterative refinement solver having either the FP32 as lower precision (using the GM refinement method) or the FP16-TC as lower precision (using the GM, IR and IRGM refinement methods). The matrix size is *n* = 22 000. Matrix of type 5: positive eigenvalues and arithmetic distribution of its singular values *σ*_*i*_ = 1 − ((*i* − 1)/(*n* − 1))(1 − (1/cond)). Similar behaviour has been observed for matrices of types 0, 1, 3 and 7 within ± 1 iteration. (*a*) Real case. (*b*) Complex case. (Online version in colour.)

**Figure 6.**
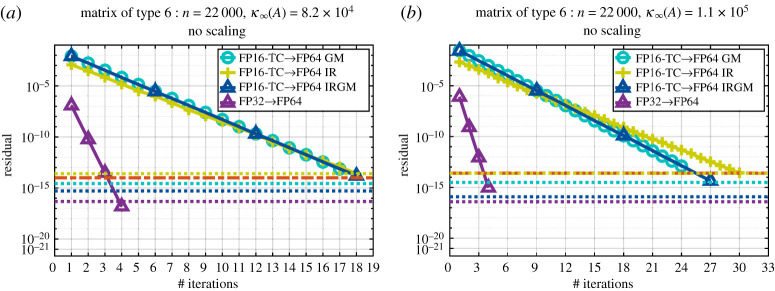
Convergence history of the proposed iterative refinement solver having either the FP32 as lower precision (using the GM refinement method) or the FP16-TC as lower precision (using the GM, IR and IRGM refinement methods). The matrix size is *n* = 22 000. Matrix of type 6: arithmetic distribution of its singular values *σ*_*i*_ = 1 − ((*i* − 1)/(*n* − 1))(1 − (1/cond)). Similar behaviour has been observed for matrices of types 2, 4 and 8 within ± 2 iterations. (*a*) Real case. (*b*) Complex case. (Online version in colour.)

Figures 5 and 6 show the convergence history given by the relative residual at each step of the refinement loop for the six proposed iterative refinement solvers (the two precision implementations— FP16-TC and FP32—each using one of the three refinement methods GM, IR or IRGM). We note that when we use the FP32 as lower precision, the three refinement methods (i.e. GM, IR and IRGM) behave exactly the same. Thus, to make graphs clear, we only show the GM implementations and denote them by FP32→FP64. The graphs are labelled as FPXX→ FP64 YY, where ‘XX’ corresponds to the lowest precision used during the LU factorization (FP16-TC or FP32), and ‘YY’ represents the iterative refinement technique (GM, IR or IRGM) used to attain the FP64 solution accuracy. In addition, in order to also compare the quality of the solution, we draw three horizontal lines that correspond to the residual of the solution at the output of our iterative refinement solver as well as the one at the output of the standard FP64 precision (dgesv and zgesv for real and complex cases, respectively).

In figure 5, we display the convergence history of a matrix of type 5, but as mentioned in the caption, we observed the same behaviour for the matrices of types 0, 1, 3 and 7 within ± 1 iteration difference. Here we can see that the tcairs using the FP32 as lowest precision converges within 3 iterations. Furthermore, we observe that the tcairs using the FP16-TC as lowest precision converges within four iterations for all refinement method variants (GM, IR and IRGM). For these type of matrices, since the number of iterations is small, we can expect a large speedup over the FP64 routine. We expect that the FP32 solver will achieve a 2× speedup and the FP16-TC one will achieve about 4× speedup while delivering a solution at FP64 accuracy. More details about the performance are provided in the next section.

Figure 6 represents a matrix of type 6, which is very similar to type 5 in the sense that they have the same singular values. However, their eigenvalues differ. We also mention that the same convergence trend has been observed for other matrices of types 2, 4 and 8. The matrices generated with types 2, 4, 6 and 8 characteristics are more difficult than the ones with types 0, 1, 3, 5 and 7. The convergence of FP16-TC requires more iterations, namely ≈17 iterations. Interestingly, the FP16-TC solver is doing well for this type of harder problem and is able to bring the solution down to the FP64 accuracy within an acceptable number of iterations. As a result, we expect from figure 4 that the FP16-TC will provide about 3–4× speedup over the FP64 counterpart solver while delivering a solution at FP64 accuracy.

**Figure 7.**
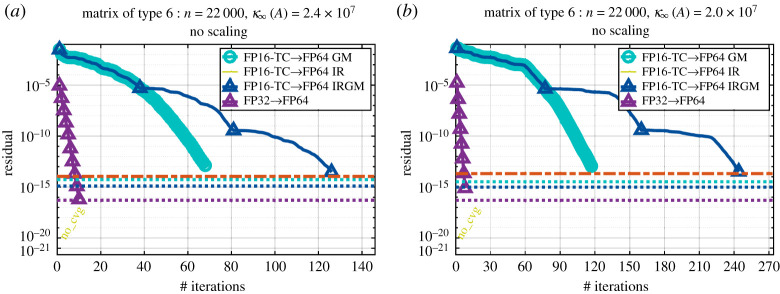
Convergence history of the proposed iterative refinement solver having either the FP32 as lower precision (using the GM refinement method) orthe FP16-TC as lower precision (using the GM, IR and IRGM refinement methods). The matrix size is *n* = 22 000. Matrix of type 6: arithmetic distribution of its singular values *σ*_*i*_ = 1 − ((*i* − 1)/(*n* − 1))(1 − (1/cond)). The matrix is generated with a large condition number to show the difference between the three proposed iterative refinement algorithms as well as to display the effect of the condition number on the convergence rate. (*a*) Real case. (*b*) Complex case. (Online version in colour.)

We mention that we performed some experiments with a basic FP16 implementation that does not use the Tensor Cores and we found that this implementation requires many more iterations to converge for this type of matrices. It also fails for other problems like the one in figure 7 and many of the sparse problems shown below. This is because the accumulation in the FP16-TC compared with the basic FP16 is done in FP32 arithmetic and thus produces a better result than the pure FP16 [30]. In addition we should not forget the fact that a basic FP16 (e.g. using the standard fused multiply–add (FMA)) will not exhibit the same high speed as the one with Tensor Cores. Such an algorithm and implementation will therefore be substantially slower, so we do not discuss it in this paper.

Figure 7 shows a substantially more difficult matrix. It is a matrix of type 5 where we increased the condition number to be within the FP32 range rather than the FP16-TC range. With such a configuration, even the FP32 will have trouble converging within three iterations, and will require about 10 iterations. For this type of matrix, and such a high condition number, the FP16-TC→FP64 IR variant is not able to converge. The basic IR method solving *Ac*_*i*_ = *r*_*i*_ directly using the computed LU factors and the solution is not accurate enough to allow the IR method to converge. However, both the IRGM and the GM refinement techniques (FP16-TC → FP64 IRGM, and FP16-TC → FP64 GM) are able to achieve the FP64 solution accuracy. This reveals the sensitivity of the IR variant and highlights the importance of using the preconditioned GMRES method. We also note that the triangle marker in the blue line—the curve that represents FP16-TC → FP64 IRGM—shows the number of outer iterations (outer refinement loop) in the IRGM solver. In our experiment, the GMRES tolerance of the IRGM method is set to 10^−4^. Thus, at each outer iteration of the refinement loop of the IRGM method the solution of *Ac* = *r* is correct to 10^−4^ accuracy. We can see that the number of outer iterations is about 4, which is consistent with the theory of [20,25]. More details about using GMRES inside the refinement process can be found in [20,25].

Now, when comparing the two successful refinement methods in figure 7 (i.e. GM and IRGM), we note that the GM method converges faster than the IRGM one. The GM method uses preconditioned GMRES to solve the whole linear system *Ax* = *b*, using the *L* and *U* factors as a preconditioner instead of using the preconditioned GMRES to solve the correction equation *Ac* = *r*, as in §6. In the IRGM method GMRES will stop at 10^−4^, then the outer refinement loop will compute a new residual, and then another GMRES is called to solve the new correction equation. Thus, the IRGM method can be viewed as a restarted GMRES—but instead of performing *m* iterations before each restart, it performs any number of iterations until it reaches the tolerance set for the GMRES (i.e. 10^−4^ in this study), and only then restarts. This is confirmed by the shape of the IRGM convergence history, and we can see that after each outer iteration (triangle in the blue line) the GMRES starts constructing a new Krylov basis to solve the new correction equation *Ac*_*i*_ = *r*_*i*_. This hard case example highlights the benefit of using a full GMRES to solve the system (e.g. the GM method) where the GMRES benefits from the entire previously constructed basis to compute the new direction for each new solution.

To make this study more general and cover many real life cases, we conducted the same experiments for sparse matrices arising from different engineering areas from the SuiteSparse Matrix Collection. The names, size and condition numbers of these matrices are given in table 5. We illustrate the convergence history results in tables 3 and 4 (the ‘no scaling’ column). These matrices have condition numbers varying from 10^1^ to 10^10^. The iterative refinement solver using the FP32 as lower precision (table 3) converges for all of these cases, except for the problem ‘*ramage02*’, where we also found that the scaling technique can be the remedy. Interestingly, we notice that the tcairs using the FP16-TC precision as the lowest precision (table 4) is able to converge for most of these problems and provide a solution similar to the one provided by the FP64 routine within a small number of iterations. These results highlight the benefit that the tcairs can bring to such applications, and one can expect a large speedup for these problems. We were surprised by the wide range of problems that the FP16-TC iterative refinement solver (tcairs) can solve. These results motivated us to look into further details and analyse why for some of these problems the FP16-TC variant had issues converging. We found that if we apply scaling to the matrix we can make the FP16-TC variant cope and solve these problems as well. This will be discussed below. We also see that the GM and the IRGM methods mostly required a similar number of iterations and both converged better than the standard IR method.

**Table 3.** Number of iterations required by the proposed iterative refinement solver having the FP32 as lower precision (dsgesv) using the GM, IR and IRGM refinement methods. Also, we show the effect of the different scaling techniques in each block-column.

			no scaling			scalar scaling			diagonal scaling				diag + scalar scaling		
name	size	*κ*_∞_(*A*)	IR	IRGM	GM	IR	IRGM	GM	*κ*_∞_(*A*^*s*^)	IR	IRGM	GM	IR	IRGM	GM
crystk03	24 696	2.09 × 10^2^	1	1	2	1	1	2	2.5 × 10^1^	1	1	2	1	1	2
crystm03	24 696	8.24 × 10^1^	1	1	2	1	1	2	7.7 × 10^0^	1	1	2	1	1	2
TEM27623	27 623	4.70 × 10^3^	1	1	1	1	1	2	2.1 × 10^1^	1	1	2	1	1	2
Goodwin054	32 510	4.49 × 10^6^	2	2	3	2	2	3	2.2 × 10^4^	2	2	3	2	2	3
thermal1	17 880	1.60 × 10^3^	1	1	3	1	1	2	8.0 × 10^1^	1	2	3	2	2	2
Zhao1	33 861	4.45 × 10^2^	1	1	3	2	1	2	1.6 × 10^1^	2	2	2	2	2	2
nd6k	18 000	3.82 × 10^2^	1	1	2	1	1	2	1.0 × 10^2^	1	1	2	1	1	2
nd12k	36 000	3.51 × 10^2^	1	1	2	1	1	2	1.0 × 10^2^	1	1	2	1	1	2
epb1	14 734	2.62 × 10^4^	2	2	4	2	2	3	4.4 × 10^4^	2	2	3	2	2	3
appu	14 000	1.02 × 10^4^	2	2	3	2	2	2	5.8 × 10^3^	2	2	3	2	2	2
ns3Da	20 414	3.79 × 10^3^	2	2	3	2	2	2	3.4 × 10^3^	2	2	3	2	2	2
mixtank new	29 957	4.69 × 10^11^	2	2	4	2	2	3	2.1 × 10^5^	2	2	4	2	2	3
e40r0100	17 281	2.23 × 10^8^	2	3	4	3	4	4	1.1 × 10^4^	2	2	3	2	2	2
bcsstk25	15 439	1.02 × 10^10^	1	1	1	1	1	2	6.2 × 10^2^	1	1	2	1	1	2
bcsstk37	25 503	2.27 × 10^9^	1	1	2	1	1	2	1.5 × 10^2^	1	1	2	1	1	2
raefsky3	21 200	5.18 × 10^11^	1	1	3	1	1	2	1.2 × 10^4^	2	2	3	2	2	2
thread	29 736	1.78 × 10^8^	1	1	1	1	1	2	1.3 × 10^3^	1	1	2	1	1	2
sme3Db	29 067	1.05 × 10^8^	15	10	9	15	11	9	3.8 × 10^7^	16	11	12	16	11	9
mult dcop 01	25 187	9.55 × 10^11^	3	2	6	3	4	4	1.5 × 10^8^	2	2	5	2	2	3
ramage02	16 830	4.52 × 10^8^	—	60	39	—	58	38	4.5 × 10^7^	11	12	15	37	28	22

**Table 4.** Number of iterations required by the proposed iterative refinement solver having the FP16-TC as lower precision (dhgesv-TC) using the GM, IR and IRGM refinement methods. Also, we show the effect of the different scaling techniques in each block-column.

			no scaling			scalar scaling			diagonal scaling				diag + scalar scaling		
name	size	*κ*_∞_(*A*)	IR	IRGM	GM	IR	IRGM	GM	*κ*_∞_(*A*^*s*^)	IR	IRGM	GM	IR	IRGM	GM
crystk03	24 696	2.09 × 10^2^	1	1	2	1	1	2	2.5 × 10^1^	1	1	2	1	1	2
crystm03	24 696	8.24 × 10^1^	1	1	2	1	1	2	7.7 × 10^0^	1	1	2	1	1	2
TEM27623	27 623	4.70 × 10^3^	1	1	1	1	1	2	2.1 × 10^1^	1	1	2	1	1	2
Goodwin054	32 510	4.49 × 10^6^	8	5	6	8	5	5	2.2 × 10^4^	5	5	6	9	5	4
thermal1	17 880	1.60 × 10^3^	3	4	4	3	4	3	8.0 × 10^1^	3	3	4	3	3	3
Zhao1	33 861	4.45 × 10^2^	3	4	4	3	4	3	1.6 × 10^1^	3	4	4	3	4	3
nd6k	18 000	3.82 × 10^2^	3	4	4	3	4	3	1.0 × 10^2^	2	2	3	2	2	2
nd12k	36 000	3.51 × 10^2^	3	4	4	3	4	3	1.0 × 10^2^	1	1	2	1	1	2
epb1	14 734	2.62 × 10^4^	3	4	4	3	4	3	4.4 × 10^4^	3	4	4	4	4	3
appu	14 000	1.02 × 10^4^	4	4	5	3	4	3	5.8 × 10^3^	4	4	5	3	4	3
ns3Da	20 414	3.79 × 10^3^	3	4	4	3	4	3	3.4 × 10^3^	3	4	4	—	—	—
mixtank new	29 957	4.69 × 10^11^	—	36	31	13	11	10	2.1 × 10^5^	8	9	8	—	—	—
e40r0100	17 281	2.23 × 10^8^	44	9	11	—	9	8	1.1 × 10^4^	5	6	6	—	—	—
bcsstk25	15 439	1.02 × 10^10^	—	—	—	1	2	4	6.2 × 10^2^	3	4	4	3	4	3
bcsstk37	25 503	2.27 × 10^9^	—	—	—	2	2	4	1.5 × 10^2^	3	4	4	3	4	3
raefsky3	21 200	5.18 × 10^11^	—	—	—	4	4	11	1.2 × 10^4^	7	6	8	6	6	5
thread	29 736	1.78 × 10^8^	—	—	—	2	2	3	1.3 × 10^3^	3	4	4	3	3	3
sme3Db	29 067	1.05 × 10^8^	—	15	9	74	13	10	3.8 × 10^7^	—	20	11	—	18	9
mult dcop 01	25 187	9.55 × 10^11^	—	—	—	—	108	62	1.5 × 10^8^	—	47	34	—	63	26
ramage02	16 830	4.52 × 10^8^	—	—	—	—	—	—	4.5 × 10^7^	—	179	83	—	—	—

**Table 5.** Performance for real life matrices from the SuiteSparse Collection and from dense matrices arising from radar design simulations. Two-sided diagonal scaling was used for the dhgesv-TC function.

				dgesv FP64	dsgesv FP32 → FP64			dhgesv-TC FP16-TC → FP64		
name	description	size	*κ*_∞_(*A*)	time(s)	# iter	time (s)	speedup	# iter	time (s)	speedup
crystk03	materials problem	24 696	2.09 × 10^2^	1.95	2	1.16	1.68	2	0.69	2.82
crystm03	materials problem	24 696	8.24 × 10^1^	1.95	2	1.16	1.68	2	0.69	2.82
TEM27623	3D electromagnetic	27 623	4.70 × 10^3^	2.68	2	1.54	1.74	2	0.87	3.08
mixtank new	CFD problem	29 957	4.69 × 10^11^	3.56	4	2.07	1.71	8	1.16	3.06
e40r0100	2D/3D problem	17 281	2.23 × 10^8^	0.81	3	0.53	1.52	6	0.39	2.07
sme3Db	structural mechanics	29 067	1.05 × 10^8^	3.33	12	2.09	1.59	11	1.13	2.94
Goodwin 054	Navier–Stokes	32 510	4.49 × 10^6^	4.17	3	2.33	1.78	6	1.30	3.20
ns3Da	3D Navier–Stokes	20 414	3.79 × 10^3^	1.29	3	0.81	1.59	4	0.48	2.68
Thermal1	thermal problem	17 880	1.60 × 10^3^	0.85	3	0.55	1.54	4	0.38	2.23
Zhao1	electromagnetics	33 861	4.45 × 10^2^	4.76	2	2.60	1.83	4	1.40	3.40
nd6k	2D/3D problem	18 000	3.82 × 10^2^	0.86	2	0.55	1.56	3	0.38	2.26
nd12k	2D/3D problem	36 000	3.51 × 10^2^	5.57	2	3.04	1.83	2	1.58	3.52
epb1	thermal problem	14 734	2.62 × 10^4^	0.53	3	0.36	1.47	4	0.27	1.96
appu	graph problem	14 000	1.02 × 10^4^	0.50	3	0.35	1.42	5	0.24	2.08
bcsstk25	structural problem	15 439	1.02 × 10^10^	0.59	2	0.39	1.51	4	0.29	2.03
bcsstk37	structural problem	25 503	2.27 × 10^9^	2.15	2	1.27	1.69	4	0.75	2.86
raefsky3	CFD problem	21 200	5.18 × 10^11^	1.33	3	0.82	1.62	8	0.57	2.33
thread	threaded connector	29 736	1.78 × 10^8^	3.30	2	1.85	1.78	4	1.05	3.14
mult dcop 01	circuit simulation	25 187	9.55 × 10^11^	2.09	5	1.27	1.64	34	1.16	1.80
ramage02	Navier–Stokes	16 830	4.52 × 10^8^	0.76	15	0.58	1.31	83	0.96	0.79
Poisson	2D Poisson problem	32 000	2.1 × 10^6^	4.01	2	2.15	1.81	10	1.23	3.33
Vlasov	2D Vlasov problem	22 000	8.3 × 10^3^	1.70	2	0.95	1.78	3	0.50	3.40

Lesson: For the matrices considered, the tcairs using the FP16-TC precision is the most powerful method in terms of performance, and it is able to cope with and solve many problems representing many engineering areas within an acceptable number of iterations. The FP32 refinement variants show a more consistent behaviour of small numbers of iterations regardless of the matrix types, except for minor cases. However, as shown in the analysis and in the performance sections, the maximum speedup that the FP32 variant can provide is less than 2× while the variant using FP16-TC can achieve 4×. These results suggest the surprising effectiveness and robustness of the FP16-TC arithmetic, showing it might be robust enough for use in HPC linear system solvers.

### (b) Scaling techniques

In §8 we described three scaling techniques for use in IR, which we denoted by scalar scaling, diagonal scaling and diagonal+scalar scaling. In this section we present experiments with these techniques. We first study these scaling approaches for the synthetic matrices described in table 2 and then we study their benefit for the sparse problems from the SuiteSparse Matrix Collection.

**Figure 8.**
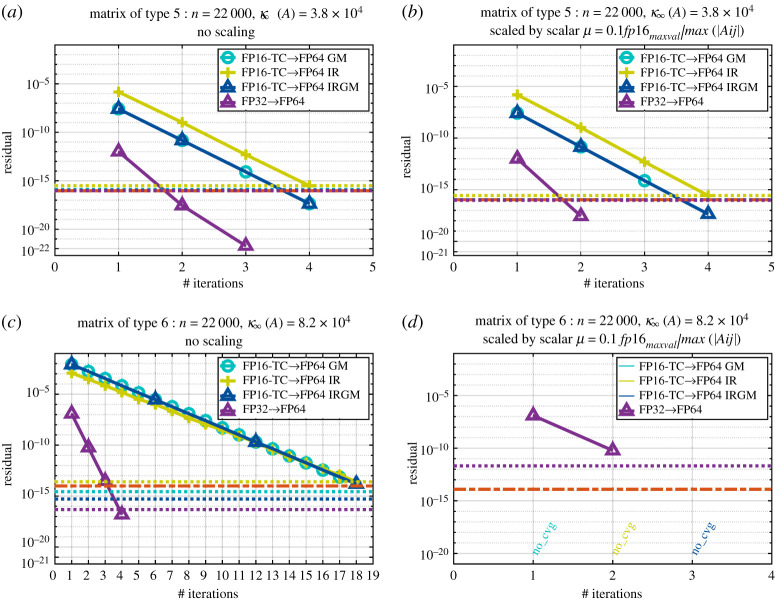
Effect of the different scaling techniques on the convergence of the iterative refinement solver. The example here, is for a matrix of type 5 (top) and type 6 (bottom) for real case of size *n* = 22 000. We mention that complex cases behave similarly. Also, matrices of types 0, 1, 3 and 7 and type 2, 4 and 8 behave similarly to type 5 and type 6, respectively. (*a*) Matrix of type 5: no scaling. (*b*) Matrix of type 5: scalar scaling. (*c*) Matrix of type 6: no scaling. (*d*) Matrix of type 6: scalar scaling. (Online version in colour.)

*Synthetic matrices.* For the synthetic matrices of table 2, we mention that only the scalar scaling need be used. The diagonal scaling technique proposed above computes a row and a column scaling intended to equilibrate the matrix *A* such that the largest element in each row and column of *A* has absolute value 1. Since the entries of the synthetic matrices are generated with a distribution on the interval [ − 1, 1], there is no need for row or column scaling. Hence we illustrate only the effect of the scalar scaling for the synthetic matrices of table 2. We choose the same matrices shown in figure 5 and figure 6 and perform the scalar scaling to the matrix *A* before we solve it using our iterative refinement solver. We illustrate the convergence history in figure 8. The graph in figure 8*a* is the convergence history of a matrix of type 5 without scaling, while the one on the right in figure 8*b* corresponds to the convergence history of the same matrix but with the scalar scaling performed. As we can see, the scalar scaling does not provide any benefit here. Already for such matrices the iterative refinement solver converged very quickly and did not suffer from any perturbation. On the graph in figure 8*c*, we represent the convergence history for a matrix of type 6 without any scaling and we depict the convergence history of the scaled matrix in figure 8*d*. Here we can see that the scalar scaling cannot improve the convergence of the three refinement methods (GM, IR and IRGM) of the FP16-TC variant, and, rather, it affects the numerics in a negative manner leading to non-convergence. The explanation for this behaviour is rather surprising: this is a rare class of matrices giving large growth factors for LU factorization with partial pivoting. Since the growth factors exceed *θ*^−1^ = 10 they cause elements of *U* to lie outside the FP16 range and then get rounded back onto the range, which reduces the quality of the LU factors, leading to non-convergence. The reasons for the large growth factors are explained in [38].

*Sparse matrices.* For the matrices from the SuiteSparse Matrix Collection we performed the three scaling techniques and illustrated the benefit of each in tables 3 and 4. The second block-column of the tables (from the left) corresponds to the scalar scaling, the third block-column corresponds to the diagonal scaling and the fourth block-column (the right-most one) corresponds to applying the diagonal+scalar technique. As can be seen, the scalar scaling behaves similarly to the no-scaling for easy problems and it helped the convergence for harder cases. The diagonal scaling technique is more robust and showed benefit for most of the cases. We can see that for problems that were already easy to solve by the iterative refinement solver without any pre-treatment, it did not slow down or affect the convergence. We also noticed how beneficial the diagonal scaling was for problems where the tcairs using the FP16-TC precision (e.g. table 4) encountered trouble converging. The diagonal scaling helped the iterative refinement solver using the FP16-TC to converge on problems that the method was not able to solve without scaling. The scaling also helped decrease the number of iterations for problems where the tcairs without scaling converged but with a large number of iterations (‘*mixtank*’). We also notice the decrease in the condition number of the matrices where diagonal scaling was applied. We show for each matrix, the condition number before and after scaling. It can be seen that when the iterative refinement solver using the FP16-TC precision did not converge (e.g. dashes in table 4), the condition number of the matrices solved was very large >10^8^, and diagonal scaling helped decrease it, thereby enhancing the convergence of the FP16-TC iterative refinement solver. Similarly, for the problem ‘*mixtank*’ for example, the FP16-TC iterative refinement solver converged within a medium number of iterations, but scaling accelerates convergence. The third strategy of scaling was to combine the two scaling techniques together. Our experiments showed that this strategy showed an unpredictable behaviour. It helped some problems but also was worse than no scaling from some others.

Lesson: Scaling techniques can enhance the convergence of the iterative refinement solver, in particular for problems with large a condition number. The best scaling technique is diagonal scaling, which equilibrates the matrix. The scalar scaling might help the convergence for many of these cases, as long as the value of *θ* is taken less than the reciprocal of the growth factor, thus *μ* requires to be carefully chosen. The diagonal+scalar scaling showed that it behaves like scalar scaling for most of the cases, except for some cases it was worse than both scaling strategies.

## 15. Performance

This section presents the performance results of our two proposed iterative refinement solvers with the FP32 or the FP16-TC as lower precision—dhgesv-TC, dsgesv and zkgesv-TC, zcgesv for real and complex cases, respectively—using the GM (GMRES) refinement method, and comparing it to the full precision reference dgesv and zgesv solvers, respectively. In all the performance figures below, we illustrate the performance in teraFLOP s^−1^ of three linear solvers for both real and complex cases:

(1) the FP64 standard solver (dgesv and zgesv for real and complex, respectively), displayed in orange colour with ‘×’,
(2) the iterative refinement solver having the FP32 precision as lower precision (i.e. *u*^*f*^= FP32) and using the GM refinement method (dsgesv and zcgesv for real and complex matrices, respectively) displayed in purple colour with ‘△’,
(3) the tcairs using having the FP16-TC precision as lower precision (i.e. *u*^*f*^= FP16-TC) and using the GM refinement method (dhgesv-TC and zkgesv-TC for real and complex matrices, respectively) displayed in cyan colour with ‘°’.

We mention that the teraFLOP s^−1^ are computed using the same formula for all solvers and all plots, *P* = (2*n*^3^/3*t*) + (2*n*^2^/*t*), where *t* is the total elapsed time of the computation, which means performance reflects the time to solution. Thus, a performance that is two times higher means the computation is twice as fast. We also note that the time of the iterative refinement solver includes all the conversion required and the iterations, meaning the solver takes a full precision *A*, *x*, *b* (e.g. FP64 *A*, *b*), and returns the solution *x* in full precision (FP64 in our experiments). The study was done for both real and complex matrices and for different matrix sizes and for different matrix types. For the two iterative refinement solvers, we also depict the required number of iterations to achieve the FP64 arithmetic solution. To make the figures fully informative of all the possible information that the reader might find interesting, we show in the right ‘*y*’ axis of the graph the condition number *κ*_∞_(*A*), corresponding to the grey dotted line.

### (a) Single right-hand side

**Figure 9.**
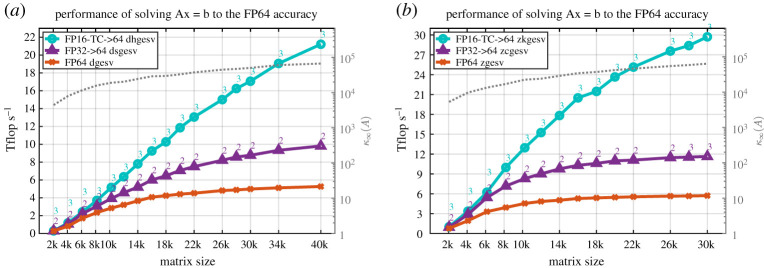
Performance in teraFLOP s^−1^ of the GM refinement algorithm for the FP32 and FP16-TC precisions studied for different matrix sizes on an NVIDIA GPU Volta GV100. Matrix of type 5: positive eigenvalues and arithmetic distribution of its singular values *σ*_*i*_ = 1 − ((*i* − 1)/(*n* − 1))(1 − (1/cond)). Similar behaviour has been observed for matrices of types 0, 1, 3 and 7. (*a*) Real case. (*b*) Complex case. (Online version in colour.)

**Figure 10.**
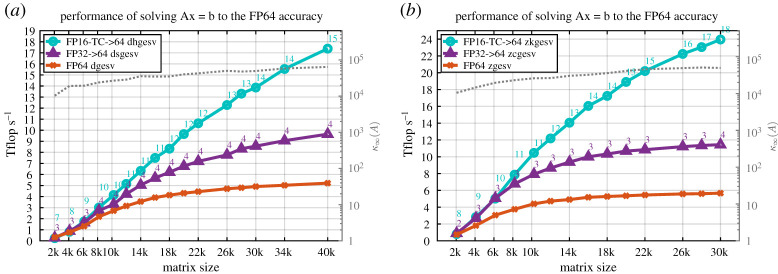
Performance in teraFLOP s^−1^ of the GM refinement algorithm for the FP32 and FP16-TC precisions studied for different matrix sizes on an NVIDIA GPU Volta GV100. Matrix of type 4: clustered singular values, $\sigma_{i}=(1,\ldots ,1,\frac{1}{\text{cond}})$. Similar behaviour has been observed for matrices of type 4. (*a*) Real case. (*b*) Complex case. (Online version in colour.)

**Figure 11.**
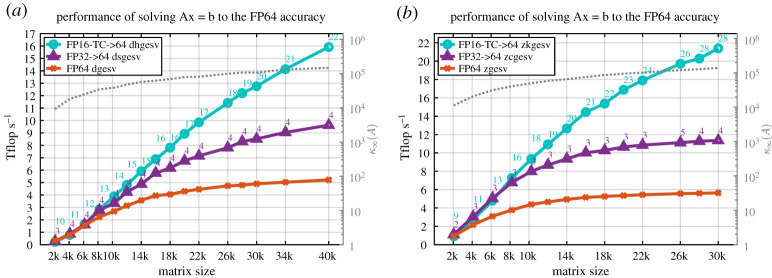
Performance in teraFLOP s^−1^ of the GM refinement algorithm for the FP32 and FP16-TC precisions studied for different matrix sizes on an NVIDIA GPU Volta GV100. Matrix of type 6: arithmetic distribution of its singular values *σ*_*i*_ = 1 − ((*i* − 1)/(*n* − 1))(1 − (1/cond)). Similar behaviour has been observed for matrices of types 2 and 8. (*a*) Real case. (*b*) Complex case. (Online version in colour.)

The performance results for the synthetic matrices of table 2 are summarized in figures 9–11. In figure 9, the matrix is of type 5, and—as shown in §14—the two precisions variants (FP32 and FP16-TC) of the iterative refinement solver require two to four iterations to converge. Thus, one can expect that the iterative refinement solver will bring a large speedup compared to its full-precision counterpart solver. Since the number of iterations is small, we presume that the speedup ratio will be similar to the one observed in figure 3*b* for the LU factorization. Our expectation is confirmed by the experimental results presented in figure 9. The tcairs using FP16-TC as the low precision (dhgesv-TC and zkgesv-TC) delivers a solution 4× and 5× faster than its FP64 counterparts dgesv and zgesv, respectively. Similarly, the iterative refinement solver variant using FP32 as the lower precision (e.g. dsgesv and zcgesv for real and complex data, respectively) shows a ≈1.8× speedup over its dgesv and zgesv FP64 precision counterparts. This example illustrates the importance of using the low FP16-TC precision in HPC. We note that our experiments showed that matrices of types 0, 1, 3 and 7 exhibited the same performance behaviour as the one illustrated in figure 9. For the real case (figure 10*a*), the iterative refinement solvers dhgesv-TC and dsgesv outperform dgesv by around 3.5 × and 1.7 ×, respectively. For the complex case (figure 10*b*), the iterative refinement solvers zkgesv-TC and zcgesv outperform dgesv by around 4.5 ×, and 1.8 ×, respectively.

In contrast to the matrices of type 5 shown in figure 9, figure 11 shows the performance and the number of iterations for matrices that have the same arithmetic distribution of the singular value, but their eigenvalues are not necessarily positive and can even be complex. This is considered a harder problem than the one shown in figure 9. For the tcairs using the FP16-TC precision as lower precision (dhgesv-TC and zkgesv-TC), the number of iterations increases compared with figure 9 (15–24 iterations vs. 3–5 iterations). We also see that the iteration count increases slightly with the matrix size. This accounts for the slightly lower speedups than in figure 9 (albeit still over 3×). The dsgesv and the zcgesv behaviour stays the same as the one shown in the previous graphs and requires about 2–3 iterations, resulting in a 1.7× speedup over the dgesv and the zgesv, respectively. The observations made here are correlated with the numerical behaviour shown in §14, figure 6. It highlights the attractive effectiveness of the FP16-TC precision and reveals the interest of using it for HPC computing. Matrices of type 2 and type 8 have shown same performance behaviour and thus we omit their graphs.

The goal of this paper is to show that the proposed iterative refinement solver can be of great benefit for a wide range of matrices with different characteristics. In practice, the real world matrices tend to be *easier* to deal with than our specially constructed synthetic ones. To illustrate this, we show results for real world matrices arising from different problems. We show in table 5 the results from experiments obtained when running three linear solvers (the standard solver dgesv and our two iterative refinement solvers using the FP32 and the FP16-TC precision, respectively) for different real world matrices from the SuiteSparse Matrix Collection. We note that the input matrices were scaled by two-sided diagonal scaling when used with the FP16-TC precision (e.g. for the dhgesv-TC function).

As can be seen, the behaviour of the proposed iterative refinement solver is similar to that of the synthetic matrices described above. For most of these problems, the iterative refinement solver using the FP16-TC as lower precision converges within fewer than 12 iterations, except for only 2 hard problems. For these hard problems, the iterative refinement solver using the FP32 as lower precision also had trouble converging within 2–3 iterations as usual. Overall, our proposed iterative refinement solver showed attractive speedup for these problems. By studying the results in the table we found that the proposed tcairs using the FP16-TC precision (e.g. the dhgesv-TC routine) can provide on average a speedup of about 2.5×–3.5× for a wide range of real world matrices and it shows about 1.7× speedup when using the FP32 as lower precision (e.g. the dsgesv routine). These results match the analysis performed in the numerical §14 and in the performance model §12.

Lesson: The speedups presented in figures 9–11 and in table 5 show that the dhgesv-TC (FP16-TC → FP64) routine can be used for most of the matrices types to provide speedups of about 3×–4×, and the dsgesv (FP32 → FP64) routine can be used for most of the matrix types to provide speedups of about 1.7×. The complex routines (zkgesv-TC and zcgesv) also showed similar behaviour as the real ones (dhgesv-TC and dsgesv) but with a slightly higher speedup over the FP64 complex routine zgesv. This is due to the higher compute intensity of the complex data computations.

### (b) Multiple right-hand sides

**Figure 12.**
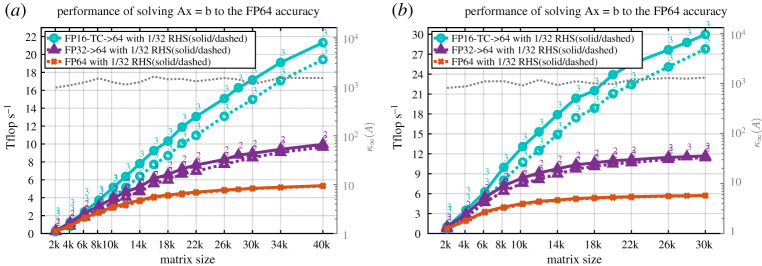
Performance of solving multiple RHSs using the three linear solvers for a matrix of type 3, for different matrix sizes on an NVIDIA GPU Volta GV100. Results are shown for 1 and 32 RHSs. (*a*) Real case. (*b*) Complex case. (Online version in colour.)

In this section we show the benefit of the tcairs when solving multiple RHSs. As discussed above, the iterative refinement solver consists of two phases: the factorization phase, which uses lower precision to achieve a high performance, and a second phase to refine the obtained solution down to the accuracy of the FP64 precision. The refinement phase is memory bound and thus it is better to converge fast (see figure 4 for the expected performance as function of the number of iterations). When multiple RHSs are needed, we cannot solve each right-hand side by itself because then we multiply the number of iterations by the number of RHSs. Thus, our strategy is to solve for all the RHSs together at once and to use Level 3 BLAS (trsm and Xgemm) during the refinement in such a way that solving for one or more RHSs will take roughly similar time (with ≈10% difference). In figure 12 we illustrate the performance obtained when solving 1 or 32 RHSs for a matrix of type 5. We see in figure 12 that solving 32 RHSs does not significantly delay the refinement process, and thus the performance obtained is only less than 10% slower than the one obtained when solving 1 RHS, for both real and complex cases. This is thanks to our proposed technique that uses Level 3 BLAS operations and optimizes the residual checking process in such a way that minimizes the amount of memory-bound operations. Interestingly, we can see that the number of iterations required to converge the 32 RHSs is similar to the one required to converge 1 RHS. These results give the tcairs an advantage even when multiple RHSs are to be solved.

### (c) Sensitivity to FP64 compute throughput

**Figure 13.**
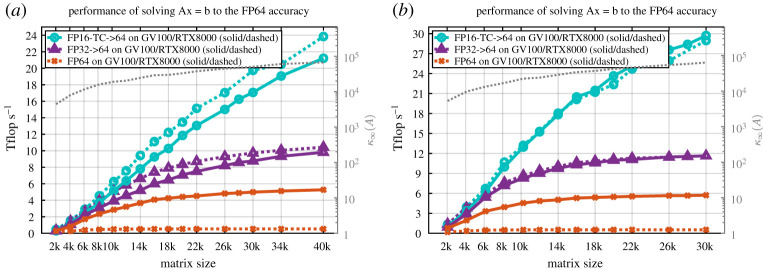
Performance comparison of the tcairs on two different GPUs: NVIDIA Quadro GV100 and RTX8000 shows lack of sensitivity to FP64 compute throughput. We illustrate the iterative refinement algorithms using either the FP32 and FP16-TC precisions as lower precision. Matrix of type 5: positive eigenvalues and arithmetic distribution of its singular values *σ*_*i*_ = 1 − ((*i* − 1)/(*n* − 1))(1 − (1/cond)). Similar behaviour has been observed for all other synthetic and real life matrices. (*a*) Real case. (*b*) Complex case. (Online version in colour.)

Based on the discussion in §10, we used our iterative refinement solver to solve the same problems studied in this paper on an NVIDIA Quadro RTX8000 (Turing TU102 GPU). Since the FP64 computations within the tcairs are used only in the memory bound refinement iterations that account for *O*(*n*^2^) of the total *O*(*n*^3^) flops, we found that the performance of the tcairs is not sensitive to the FP64 compute throughput and maintains the same high performance behaviour. Figure 13 depicts the performance obtained for a matrix of type 5 using the three solvers—the FP64 solver (dgesv or zgesv for real and complex cases, respectively), the iterative refinement solver solver with FP32 as lower precision (dsgesv or zcgesv for real and complex cases, respectively), and the iterative refinement solver using the FP16-TC precision as lower precision (dhgesv-TC or zkgesv-TC for real and complex cases, respectively)—on the GV100 and the RTX8000. We can easily see that the solver maintains the same effective FP64 performance on both GPU architectures despite their vastly different FP64 compute throughput (7 TFLOP s^−1^ on GV100 and 500 GFLOP s^−1^ on RTX8000). Here we show only one graph of performance, but all the other graphs show a similar performance trend.

## 16. Energy efficiency

**Figure 14.**
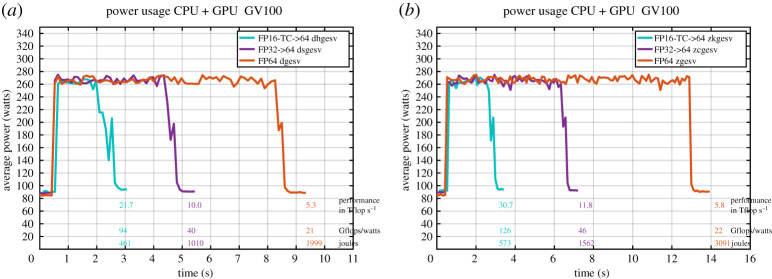
Power usage of the CPU and an NVIDIA GPU Volta GV100 for the Tensor Cores Accelerated Iterative Refinement Solver for the FP32 and FP16-TC precisions for a real and complex matrices of size *n* = 40 000 and *n* = 30 000, respectively. (*a*) Real case. (*b*) Complex case. (Online versio2n in colour.)

Figure 14 shows the energy efficiency results in real (left) and complex (right) precision, respectively. We report the sum of the CPU, DRAM and GPU power measurement. The power of the CPU (package + DRAM) is measured using the Performance Application Programming Interface (PAPI) [39], and the power of the GPU is measured using the NVIDIA Management Library (NVML) [40]. We note that the solver is GPU-only (i.e.does not use the CPU), but we still add the CPU’s idle power consumption, which is about 20–30 Watts. The standard dgesv solver provides an energy efficiency of 21 gigaFLOP s^−1^ per Watt. The FP32 iterative refinement solver (dsgesv) doubles the energy efficiency, increasing it to 40 gigaFLOP s^−1^ per Watt. This follows our performance analysis described above, since dsgesv is about twice as fast and thus we can observe twice the energy efficiency using the dsgesv routine. The most pronounced result is achieved by the FP16-TCiterative refinement solver (dhgesv-TC). It achieves an unprecedented energy efficiency of 94 gigaFLOP s^−1^ per Watt, which is about a 4.5 × improvement over the standard dgesv solver. The complex case achieves 126 gigaFLOP s^−1^ per Watt, which is more than a 5 × improvement over the standard zgesv solver. These results demonstrate that the iterative refinement methods and half-precision arithmetic will be decisive in helping mitigate the power constraints in large-scale HPC systems.

The different phases of the computation can be identified on the power graph, as we now explain. The portion that consumes very high power (e.g. 260 Watts) corresponds to the factorization phase. 90% of the factorization phase consists of compute intensive operations and thus the power will be at the peak. We can also see here that this portion that corresponds to the factorization in FP16 (cyan curve) is about 4 times faster than its corresponding one in FP64 (orange curve). Once the factorization is done, the code synchronizes all streams, and performs some checking. This corresponds to the lowest quick spike down. Then, for the standard FP64 solver (e.g. dgesv), it performs the solve phase (one solve of *LUx* = *b*) to compute the final solution *x* while for the iterative refinement solver, it performs the refinement phase (which correspond to as many solve and mat-vec as the number of iterations). The solve or refinement phase consists of memory bound operations and we know that such operations do not consume the peak power but rather they consume lower power which corresponds to the curves around 180 Watts. Thus we can easily illustrate the refinement phase in these graph by the portion that consumes around 180 Watts. We can see that the refinement portion for FP32 dsgesv is slightly shorter than the one for FP16 dhgesv-TC. This is normal since the dsgesv required about 3 iterations while the dhgesv-TC required about seven iterations.

## 17. Conclusions and future directions

Designing and implementing numerical algorithms that efficiently exploit current highly parallel computer architectures is a challenge, especially if close to peak performance is to be achieved. Nevertheless, we have developed a new class of iterative refinement solvers and a number of computational techniques that allow us to solve fundamental *Ax* = *b* problems not just close to peak FP64 performance, but to get multiple times over the peak, by using the fast mixed-precision arithmetic Tensor Cores available in new GPU architectures—all while retaining the numerical stability of an FP64 solution. In particular, we showed that the new iterative refinement solver can accelerate the solution 4- to -5× and have 5 × better energy efficiency on NVIDIA Volta GV100 GPUs. The complex case achieves an unprecedented energy efficiency of 126 gigaFLOP s^−1^ per Watt, which is more than a 5× improvement over the standard FP64 solver. The new developments further improves previous efforts in this direction, including performance, energy efficiency and applicability to real problems. The solvers are now released in the vendor-optimized numerical library cuSolver [1] from NVIDIA and the MAGMA [2] open-source numerical library.

The developments open up directions for future work, including further optimizations, development of a full set of mixed-precision factorizations, linear system solvers as well as eigensolvers and singular value decomposition (SVD), and release as open-source software through MAGMA [2]. See [41] for a mixed-precision algorithm for symmetric positive definite systems and [42], [41] for mixed-precision algorithms for the least squares problem. Furthermore, the developments illustrate that mixed-precision techniques can be of great interest for linear solvers in many engineering areas. Such methods can also be easily ported to large-scale distributed or multi-GPU environments and supercomputers, where the speedups are expected to remain the same as for a single GPU.

## Footnotes

1 Formerly known as the University of Florida Sparse Matrix Collection.

## Data accessibility

The software can be found at https://developer.nvidia.com/cuda-downloads. This article has no additional data.
